# Normal stress distribution in built-up cold-formed column in relation to interconnecting bolt spacing

**DOI:** 10.1038/s41598-024-55986-7

**Published:** 2024-03-05

**Authors:** Patryk Deniziak, Elżbieta Urbańska-Galewska, Małgorzata Gordziej-Zagórowska

**Affiliations:** Department of Civil and Environmental Engineering, Gdansk University of Technology, Gdansk, Poland

**Keywords:** Engineering, Materials science

## Abstract

In order to increase a stiffness of cold-formed steel (CFS) elements it is practised to built-up the cross-section. In the analysed case, a main element is strengthened by adding extra chord in contact partially along the column. This additional chord acts as a longitudinal stiffener connected with the main section by series of bolts. Authors check whether rules applied over the years, for hot-rolled elements, can be indiscriminately used in the analysed CFS element. The aim of this study is to experimentally and computationally recognize the normal stress distribution in axially compressed CFS built-up column chords and to evaluate the element load-bearing capacity.

## Introduction

Structure mass and costs optimization often results in thin-wall elements applications like cold-formed members. Initially, cold-formed steel (CFS) elements were used by the construction field of industry as secondary elements such as window frames, steel sheeting or purlins. Now the CFS elements are successfully practised as primary structural parts with an undiminished safety level^1,2^. Cold-formed elements are not only used as a part of industrial buildings. Those profiles can be also successfully applied in the housing industry^3,4^. Currently, numerous studies confirming the high usability of CFS members are available.

**Figure 1:**
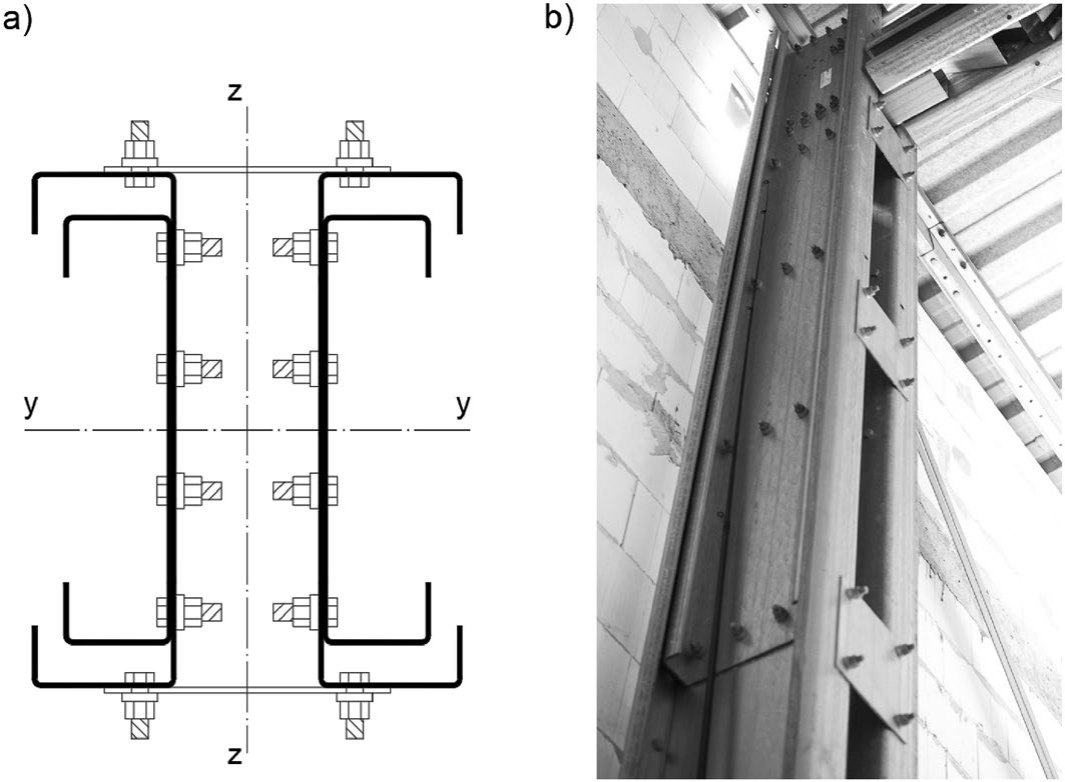
Built-up column with chords in contact and extra battens, (**a**) cross-section, (**b**) existing structure.

Due to CFS elements both global and local (plate) high slenderness, complex forms of stability loss are more likely to appear compared to hot-rolled equivalents. In order to increase the stiffness, designers strengthen members by cross-section expansion and adding extra chords in contact. To improve stability to mass radio, it is reasonable to built-up the element (by adding closely-spaced chords) only in part of the column length. This solution has its practical application in some existing steel halls constructions. An example of an actual built-up column is shown in a Fig. 1. In this case, an additional (internal) chord acts as a longitudinal stiffener connected by series of bolts.

According to the Eurocode^5^, maximum spacing for interconnections should not exceed 15 i_min_, where i_min_ is the minimum radius of gyration of one chord. Satisfying this condition allows the column to be treated as a single integral member. Authors check whether rules applied over the years can be indiscriminately used in analysed CFS element.

Over the last few years number of publications on the load-bearing capacity and stability of built-up thin-walled elements has increased. The article^6^ describes behaviour of an innovative built-up cross-section for compression elements with the use of double C-sections with extra chords inside the main ones. This solution was successfully used during the existing building restructuring. Experimental results^7^ show a significant relationship between buckling resistance and the presence of additional cross-section strengthening of the compressed closely-spaced bars. Another study^8^ shows experimental analysis of cold-formed C-section steel compression member partially stiffened along the element. Authors present double back-to-back channel cross-section strengthen with extra chord in contact variating its length. Results show that the additional chord length increase improves the load capacity of the element and the failure mechanisms may occur in the main section beyond the range of the expanded section.

Laboratory tests on the built-up cold-formed laced section are presented in articles^9–11^. Test results relate to American, Australian and European code calculations in the context of stability loss mechanism and final resistance. Authors inform that codes accurately predicted local buckling modes but procedures are less precise in the field of global buckling behaviour. Similar tests described in^12^ indicate Eurocode as a more precise code in the case of built-up column behaviour. Authors of^13^ presented a parametric study and improved design guidelines for CFS battened built-up columns.

The authors in^14,15^ present experimental research on back-to-back screw connected CFS C-sectional column. It was found that both local and global forms of stability are not highly dependent on fasteners location. T-shape columns experimental investigation^16^ shows that variation in bolt spacing had minor effect on the element resistance. Similar cross section was analysed in^17^. Research shows that the use of more screw rows does not substantially improve the ultimate column capacity. The conclusions were supported by numerical analyses as well. Research described in^18^ confirms that built-up cold-formed column capacity is not greatly dependent on bolt spacing.

Many papers confirm the legitimacy of FE method in designing process of CFS sections. The authors in^19^ presented the study on back-to-back Sigma CFS column. Members with different slenderness were checked according to experiment and computational analysis. Good consistency between FEM model results and experimental ones at the level of 95% has been achieved. Article^20^ presents high compliance of computational build-up model and an experiment. After model validation it is possible to predict the behaviour of the elements from outside the experimental set. Due to the possibility of contact implementation, it is possible to model closely-spaced elements^21–24^. The high sensitivity of shell models to given imperfections was presented in works^25–27^, where the size and form of imperfections significantly affect the obtained results^28,29^.

Mutual cooperation of closely-spaced CFS elements, although used by the construction industry, is not sufficiently recognized and still worth investigating. The biggest motivation for this paper analysis is the lack of the experimental and computational investigation for double C-section CFS compressed columns with an additional closely-spaced chord (located fragmentarily inside the main one) connected by series of interconnecting bolts.

## Experimental research

### The aim of the research

**Figure 2:**
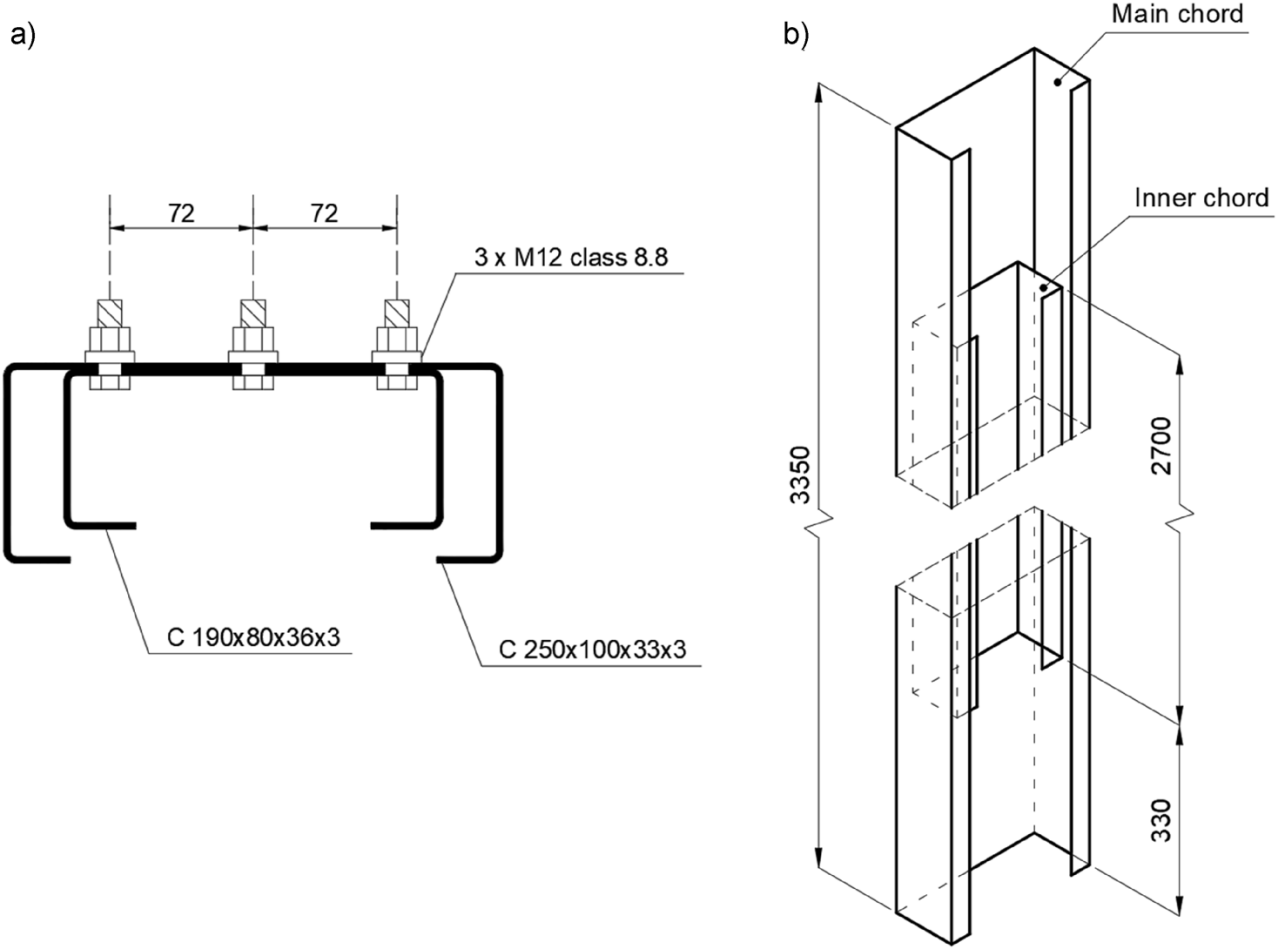
Analysed build-up column, (**a**) closely-spaced cross-section, **(b**) mutual location and lengths of the individual chords of the built-up column.

The aim of the laboratory research was to identify the normal stress distribution along the chords of axially compressed column (Fig. 2), depending on the spacing of interconnecting bolts series. The second goal was to determine the maximum load-bearing capacity of analysed element. Level of column strengthening by taking into account the presence of an internal stiffener was also described.

### Research programme

Used by the industry, closely-spaced built-up cross-section was selected as an axially compressed test object consisting of two cold-formed C-sections. The thickness of those elements was equal to 3 mm. The main chord was the C 250 × 100 × 33 × 3. Internal stiffening chord was made out of C 190 × 80 × 36 × 3 section. The profiles were interconnected by a series of M12 bolts (class 8.8) located along the element—three bolts in each row. Figure 2a shows analysed closely spaced built-up column cross-section.

Length of the main chord was 3350 mm. The internal chord (2700 mm in length) was located symmetrically about the column. The mutual location and lengths of the individual chords of the built-up column are shown in Fig. 2b. The assumed schematic scheme was double hinged.

**Figure 3:**
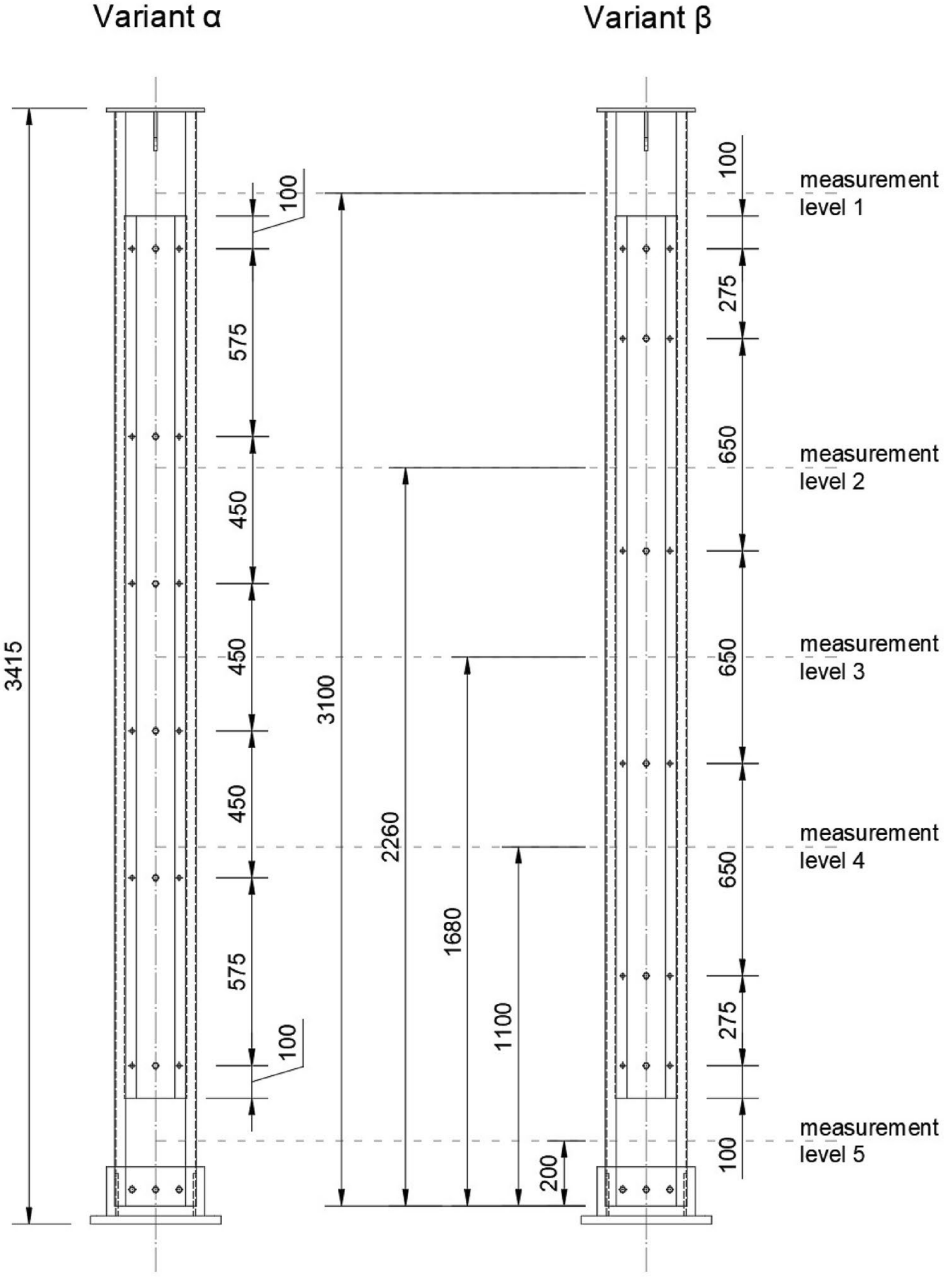
The spacing of the interconnection bolt series in both variants and location of measurement levels.

Two variants of columns were experimentally analysed. The spacing of the bolt series was varied but the number of total interconnecting bolts in both variants were equal. Seven built-up columns were compressed during the test session. The first group, called an α variant, had a bolt spacing based on the Eurocode practise defined for hot-rolled sections. EN 1993-1-1^5^ recommends to apply the maximum bolt spacing as mentioned $15 \cdot i_{min}$. In the analysed case required spacing translates to 450 mm. The second group, called a β variant, referred to extended bolt spacing to a value equal to 650 mm (44% increase comparing to the α variant). Figure 3 presents the spacing of the interconnection bolt series in both variants and location of measurement levels, which are understood as cross-sections of the tested element in the horizontal plane, located at fixed heights in relation to the lower edge of the column section. The concept of measurement levels was introduced to present the state of stresses and strains in the tested element.

Single column consisting of the main chord only was also tested in order to verify its load-bearing capacity in a laboratory manner and to set a reference point for the built-up models.

The column models ordered from the manufacturer were delivered to the laboratory with all bolts previously tightened to a controlled torque of 70 Nm.

### Research station

**Figure 4:**
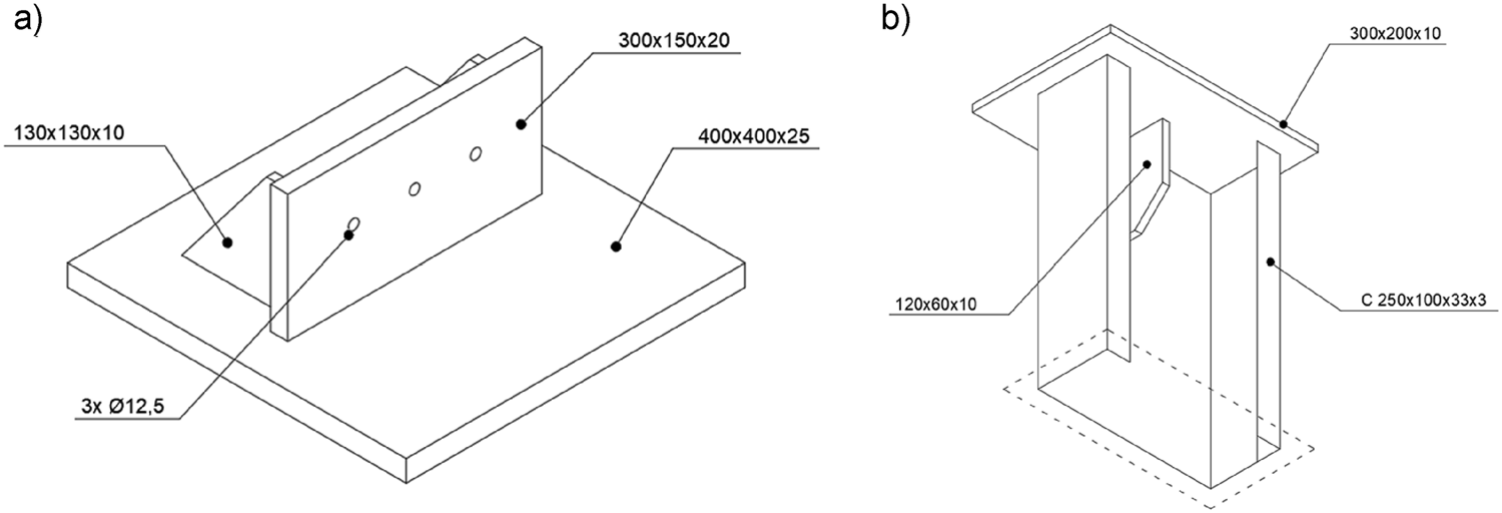
Shape and dimensions of, (**a**) column base, (**b**) column head.

The lower support of the tested elements was a welded base consisting of a horizontal plate transmitting loads to the foundation and a vertical plate with holes, to which the thin-walled main chord was connected. Additionally, two transverse stiffeners were added to provide adequate stiffness to the entire base. In order to evenly transfer the force from the main chord to the horizontal sheet, the bottom edge of the column was also supported. The shape and dimensions of the base are shown in Fig. 4a.

At the top of each column, there was a 10 mm thick horizontal plate welded on. The shape and dimensions of the column head are shown in Fig. 4b. The presence of a horizontal plate and a transverse stiffener additionally stiffened the cross-section in the support area.

**Figure 5:**
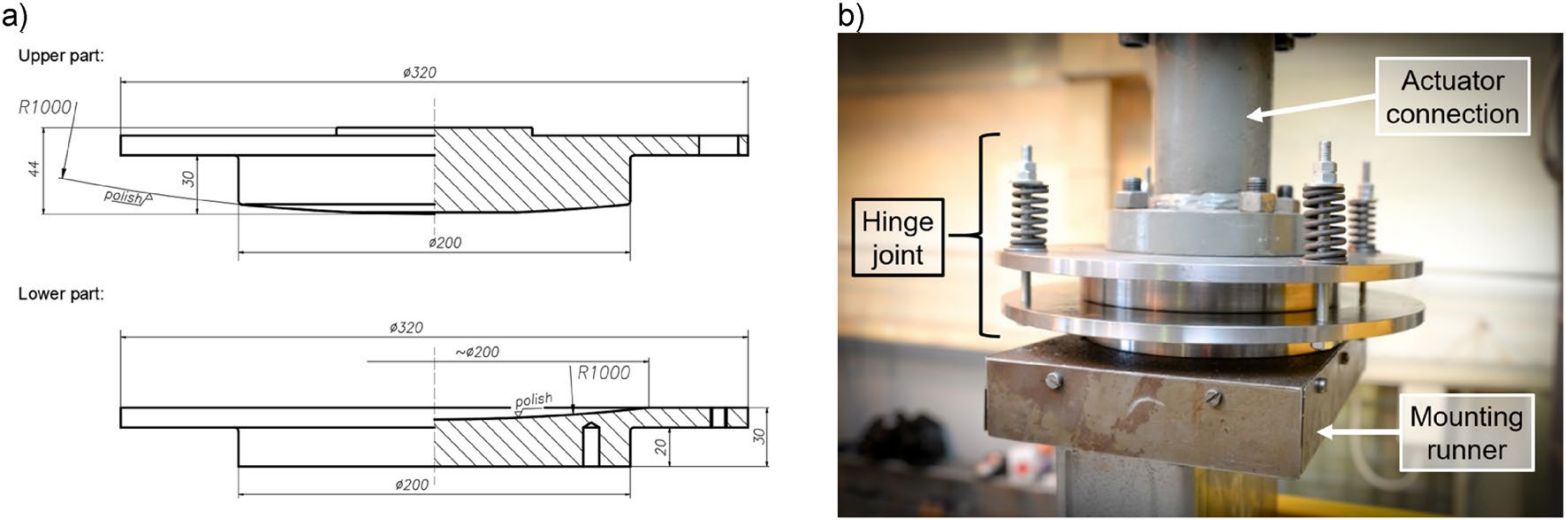
Column head spherical hinge, (**a**) cross-section, (**b**) photo of mounted hinge on the research station.

A top spherical hinge joint, designed and made for the needs of this experiment, was placed on the horizontal plate. A cross-section through the joint structure is shown in Fig. 5a. The hinge was made out of S355 steel and consisted of two steel blocks with circular surfaces sliding on each other. The components of the joint were made using a high-class computer-controlled CNC milling machine type HAAS EC-1600 with a surface accuracy of 5 µm. The use of spherical hinge ensured the compression load flowed axially at each stage of the experiment regardless of column deformation. A photo of the complete column head on the research station is shown in Fig. 5b.

Both at the design and assembly stage of the research station, great attention was paid to the mutual position of the head elements. The centres of gravity of the actuator, dynamometer, spherical hinge joint and the horizontal plate of the head were located in one vertical line, which was an extension of the axis of gravity of the column main chord itself. Furthermore, the location of built-up section centre of gravity coincides with the centre of gravity of the single main section. Consequently, no additional eccentricity occurred.

Linear gauges were located at 5 measurement levels at different heights. In addition, one strain gauge was used to take into account temperature changes during the test. The placement of the measurement levels, where the displacement sensors and strain gauges were located, was constant for columns in both variants. The view of the tested element with the base and the head as well as the marked measurement levels are shown in Fig. 3. The displacements were measured by mechanical sensors located on the first and third measurement levels.

**Figure 6:**
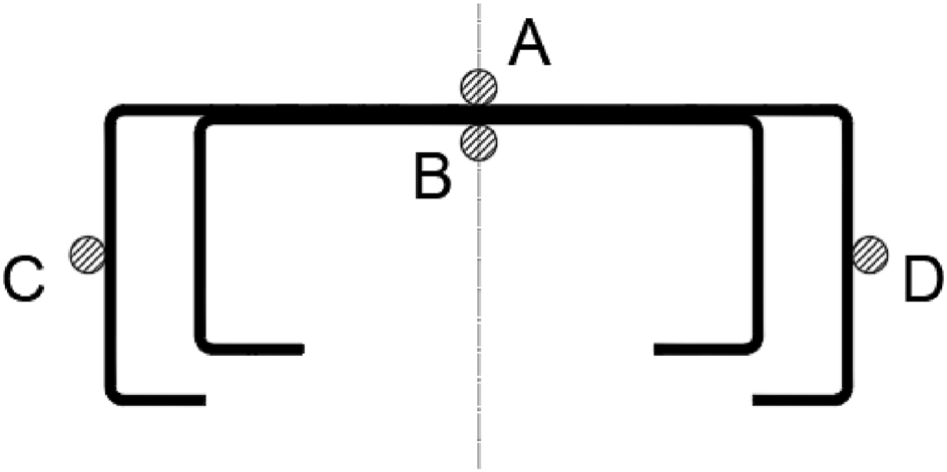
The location of measurement points on the contour of the built-up cross-section.

There were 4 points marked on the contour of the built-up column, named successively with letters: A, B, C and D, which enabled an unambiguous description of the measurement locations. Figure 6 shows the location of these points in the cross-section.

**Figure 7:**
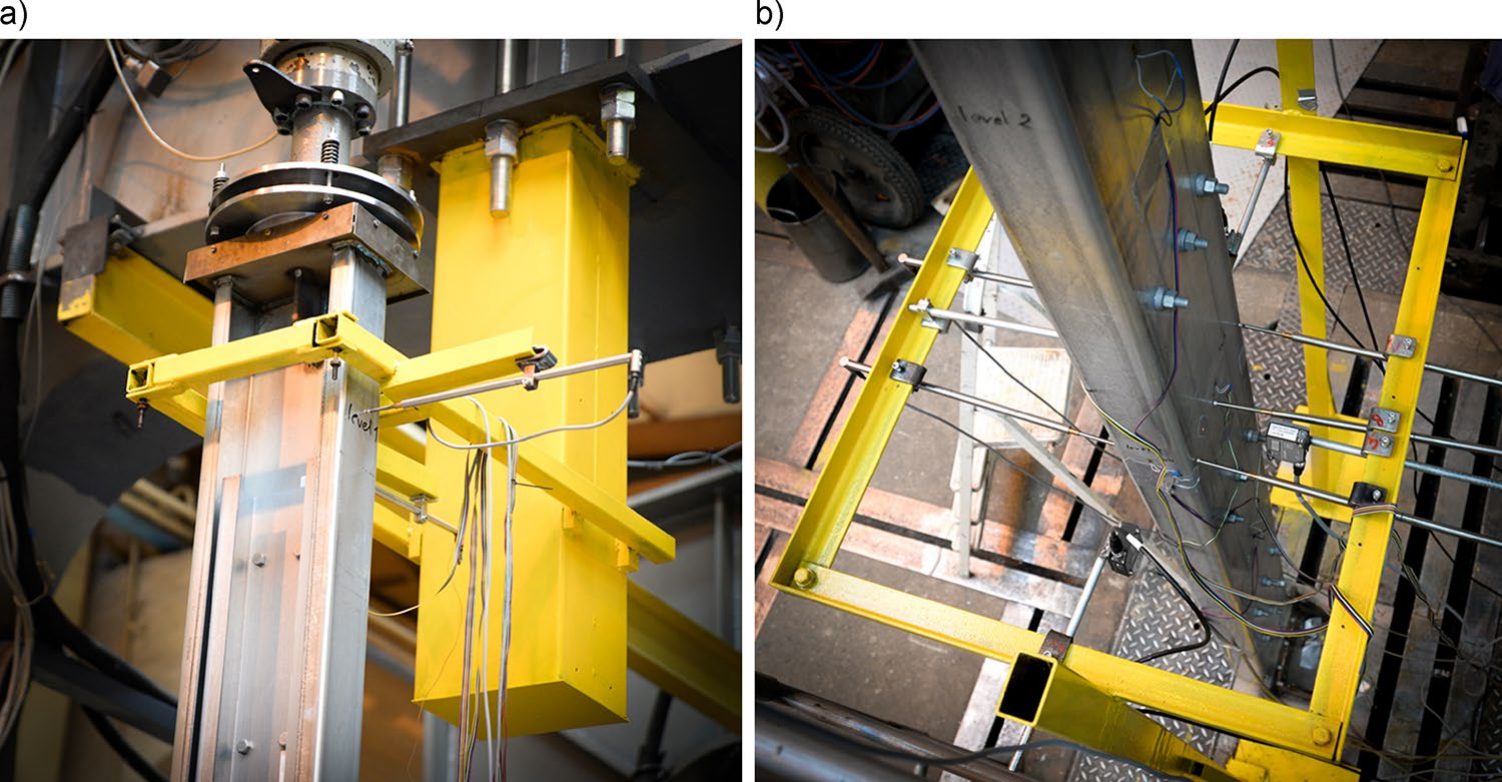
Photo of sensors location and mounting at, (**a**) level 1, (**b**) level 3.

Table 1 presents the location of strain gauges and displacement sensors in relation to the measurement levels shown in Fig. 3. The discussed system was applied to both bolt spacing variants (α and β). Figure 7 shows the location of sensors at levels one and three.

**Table 1:** Location of strain gauges and displacement sensors in relation to the measurement levels.

Measurement level	Strain gauges location	Displacement sensors location
Level 1	1 Strain gauge on the web (A*)	2 Displacement sensors (A + D*)
Level 2	2 Strain gauges on the web (A + B*)	No displacement sensors at this level
Level 3	4 Strain gauges on the web (A,B,C,D*)	5 Sisplacement sensors (acc. Figure 7b)
Level 4	2 Strain gauges on the web (A + B*)	No displacement sensors at this level
Level 5	1 Strain gauge on the web (A*)	No displacement sensors at this level
**Total number of sensors in single model along its height**	**10 pcs + 1 self-compensated strain gauge**	**7 pcs**

*According to the Fig. 6

## Research results

### Material tests

Material tests were carried out in accordance with the EN ISO 6892^30^. The aim was to determine strength parameters of the material and to identify the exact stress–strain relationship for the steel used in this research.

**Figure 8:**
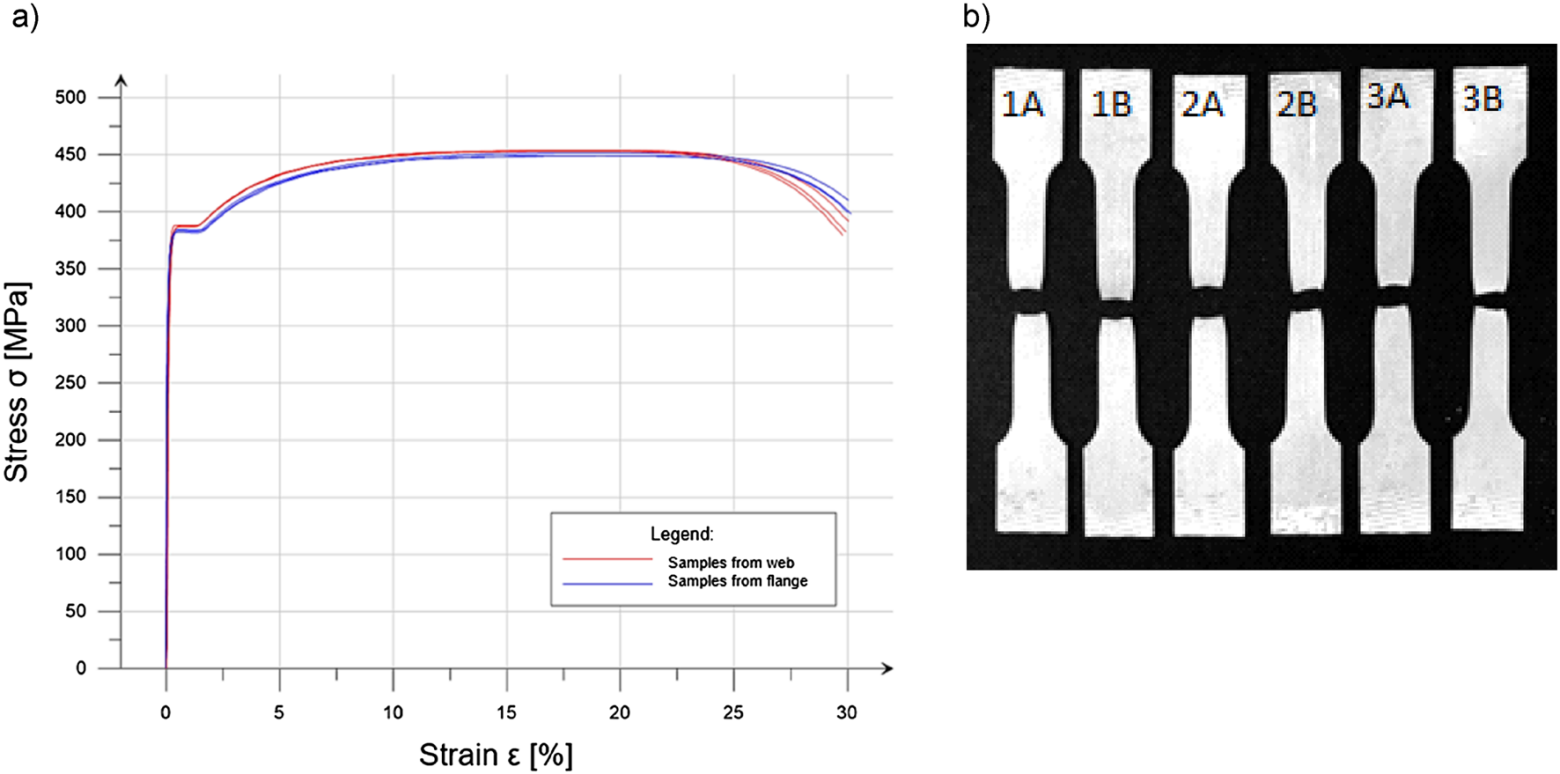
Results of material test, (**a**) stress–strain relationship for analysed samples, (**b**) steel samples after failure.

Flat samples with a rectangular cross-section were used. The tensile samples had heads for easier assembly in the testing machine and to control the distribution of stresses along the length of the element. The test samples were obtained from both the flanges and the web from the chord that was not intended for the column compression experiment. The samples were marked according to the scheme: number and letter (for example "2B"). The number represents the order of the samples in the test. The location of sampling was marked with a letter: A—sample taken from the web, B—from the flange. The assumed steel was S350 GD + Z. Test results are presented in Table 2 and Fig. 8a. Figure 8b shows steel samples after failure.

**Table 2:** Summary of material tests for samples obtained from a thin-walled column.

Sample symbol	Initial cross-section area	Max tensile force	Ultimate stress	Yield stress	Modulus of elasticity
	$S_{0}$ (mm^2^)	$F_{m}$ (kN)	$\sigma_{u}$ (MPa)	$\sigma_{y}$ (MPa)	E (GPa)
1A	60.0	27.2	454	388	215
1B	60.0	27.1	451	383	178
2A	60.0	27.2	453	388	167
2B	60.0	26.9	449	384	225
3A	60.0	27.2	453	387	215
3B	59.85	26.9	449	382	219
**Average value**	**–**	**27**	**452**	**385**	**203**
Standard^1^ value for S350 GD + Z	–	-	420	350	210

Averaging the results of 6 measurements, the yield strength was 10% higher than the standard value. In the case of ultimate strength, it was noticed to be 8% higher than the standard value.

In order to additionally confirm that the steel of material samples belongs to the S350GD + Z grade, their quantitative chemical composition analysis was carried out. A spark emission spectrometer was used. This research is generally based on the analysis of the electromagnetic radiation spectrum of the tested metal subjected to the excitation. The obtained results were consistent with the standard^31^.

### Axially compressed built-up columns

The test was controlled by a constant increase in the vertical displacement. The columns were loaded until the force value read from the dynamometer decreased explicitly. Three columns in the α variant (test no. 1–3) and four columns in the β variant (tests no. 4–7) were examined.

At the end of the series of experiments, one additional column without an internal, closely-spaced chord was tested. The aim was to experimentally test its load-bearing capacity and evaluate the sense of cross-section building-up (test no. 8). In order to obtain comparable results, the eighth column was supported in the same way as previous 7 models. Except for the lack of an internal chord, the geometry of the column itself, as well as the head and base, was unchanged.

Table 3 shows the experimental results of built-up column compression tests. A high consentience of the results obtained in tests 1 to 7 was noted. The three-sigma rule was used as a statistical test criterion for rejecting a single erroneous measurement^32,33^. Test results were within the mentioned standard deviation range, so no result was discarded.

**Table 3:** Experimental results of built-up column compression tests.

	Max vertical force P_max_ (kN)	Averaged vertical force P_avg_ (kN)	Standard deviation: s (kN)	Median (kN)
Variant α				
Test 1	320.20	319.44	13.67	320.20
Test 2	332.71			
Test 3	305.40			
Variant β				
Test 4	333.74	333.80	18.03	327.52
Test 5	359.31			
Test 6	321.30			
Test 7	320.83			
Test 8*	242.27	242.27	-	242.27

*Additional test – column consists only on external chord.

The value of the load capacity of a column consisting of the main chord only (test 8), although based on one laboratory test, corresponded with Eurocode calculations (experiment 242 kN, Eurocode^1,5,34^: 227 kN). Load bearing capacity increase of 35% was noticed comparing single chord column (test 8) to built-up columns (average from tests 1–7).

Table 4 shows the load-bearing capacity of single and built-up cold-formed sections based on Eurocode^1,5,34^. The main cross-section capacity is equal to 364 kN and built-up element capacity is 388 kN (Table 4). Calculated values are greater than experimentally achieved results due to stress concentration in the bottom area of the column. In analysed case the column resistance is conditioned by cross-sectional resistance near the bottom end.

**Table 4:** Load-bearing capacity of single and built-up cold-formed sections based on Eurocode^1,5,34^

	C 250 × 100x33 × 3	C 190 × 80x36 × 3
Cross-section class according to^5^	IV	IV
Cross section capacity (local and distortional buckling included)^35^	364 kN	345 kN
Element capacity (global, local and distortional buckling included)^35^	227 kN	205 kN
Built-up element capacity (global, local and distortional buckling included) as a uniform element along the entire length of the column	388 kN	

**Figure 9:**
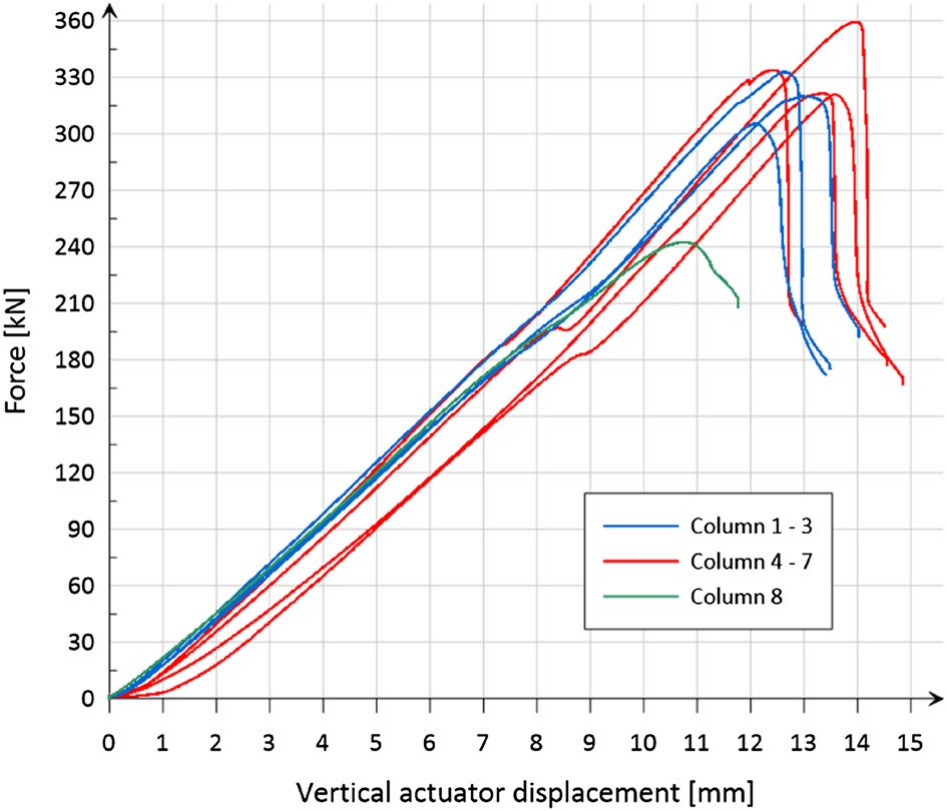
Relationship: applied force—vertical displacement of the actuator.

Figure 9 shows the dependence of the applied force on the vertical displacement of the actuator for all eight tested models. A consistent character of the curves was noticed for columns 1–3 (variant α) and 4–7 (variant β).

**Figure 10:**
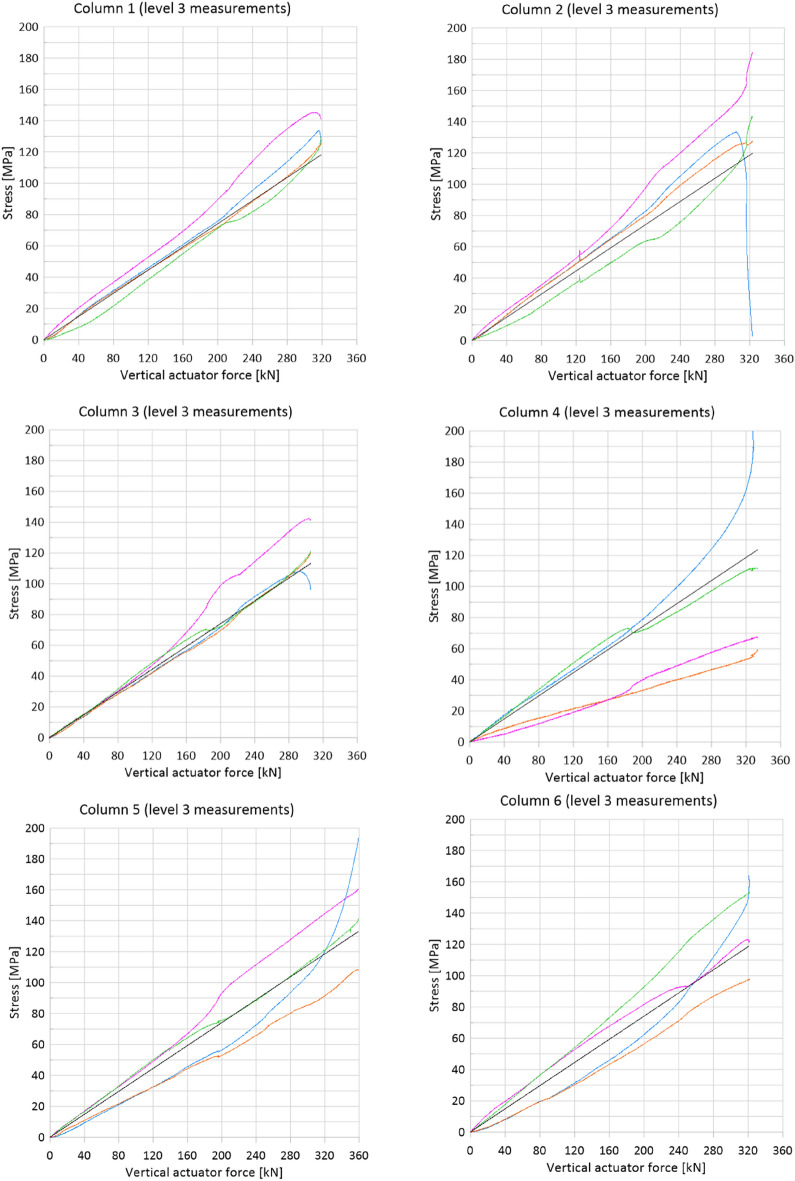

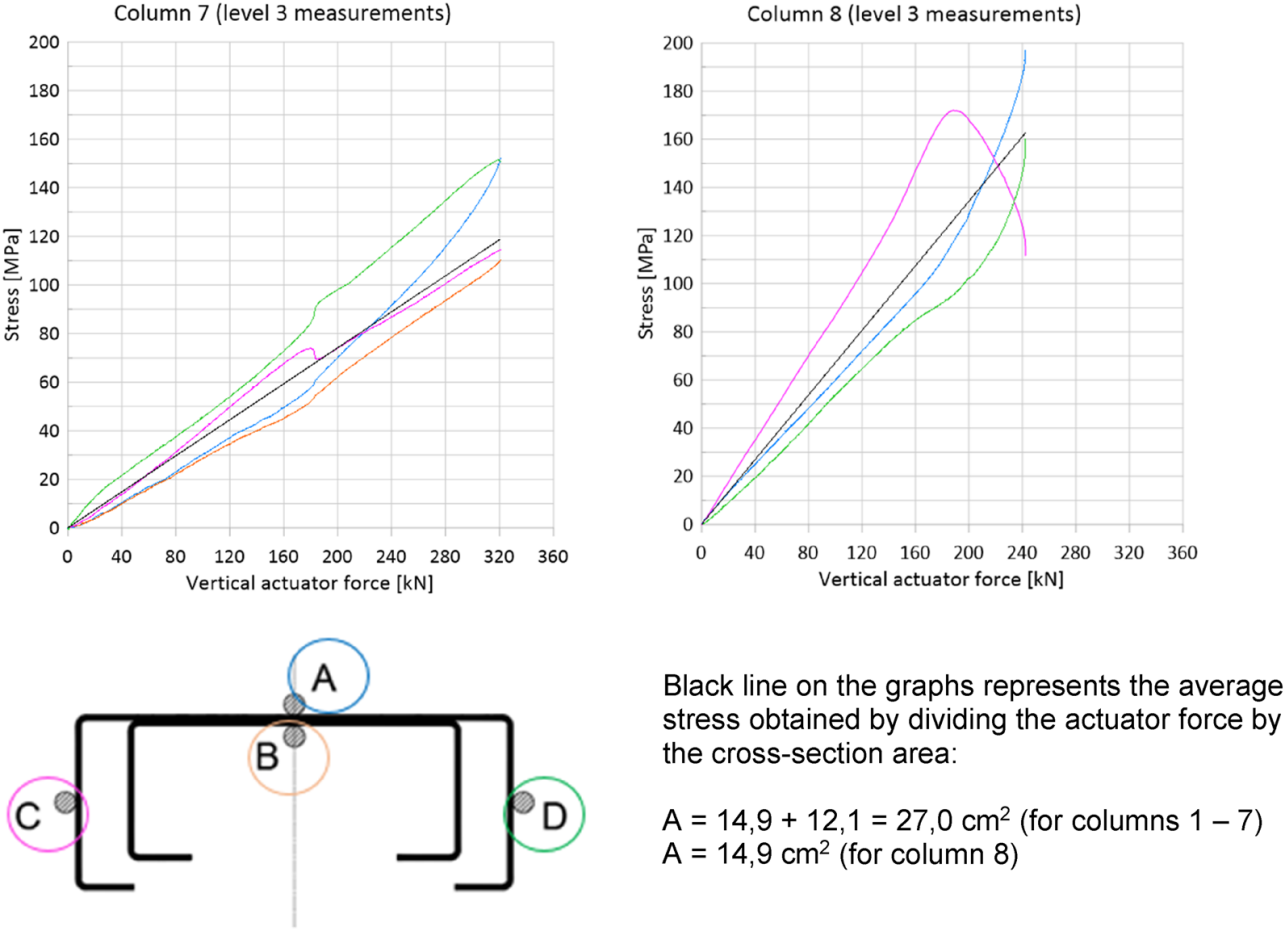
Dependence of normal stresses on the vertical actuator forces observed at level 3.

Values read from strain gauges were converted into stresses. It was noticed that the measured strains correspond to stresses entirely in the elastic range (much below the yield strength equal to 385 MPa). Therefore, a linear conversion factor was used. The modulus of elasticity E was assumed to be 203 GPa—the value obtained from the tests of material samples (Table 2). Figure 10 shows the dependence of normal stresses on the vertical actuator forces observed at level 3 (Table 1). The consistent strain distribution over the entire cross-section of the main chord at level 3 was observed which confirmed the assumption of column axial compression.

**Figure 11:**
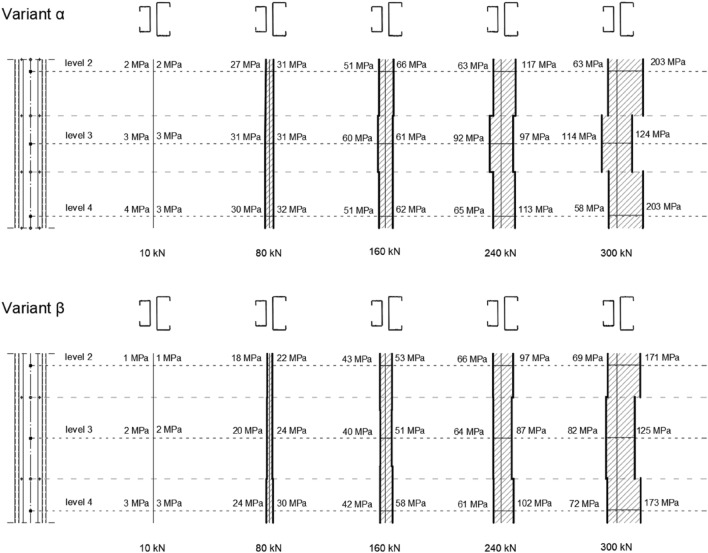
Stress diagram showing the distribution of normal stresses parallel to the column axis along the length of the tested element for the vertical load of 10 kN, 80 kN, 160 kN, 240 kN and 300 kN.

Based on the taken values from the group of strain gauges located on the webs of both the main and the additional chords, a stress diagrams were made showing the distribution of normal stresses parallel to the column axis along the length of the tested element. In order to graphically interpret the flow of stresses between the main chord to the additional one, it was assumed that the stress values are constant between the bolt series. Diagrams showing averaged web stress distribution for each variant along the length of the tested column with an axial load of 10 kN, 80 kN, 160 kN, 240 kN and 300 kN are shown in Fig. 11. The compiled stress distributions diagrams show, that the internal chord takes a significant part of the stresses in both experimentally tested variants.

Displacement measurements were analysed on two levels (2 displacement sensors on the first level and 5 sensors on the third level). The layout of displacement sensors is presented in Table 1.

The horizontal movement of the column head was monitored using two mutually perpendicular spring displacement sensors (Fig. 7a). The readings were taken in the plane 25 cm below the horizontal plate of the head (measurement level 1). When analysing the data from sensors near the column head, deformation of no more than 3 mm was found.

**Figure 12:**
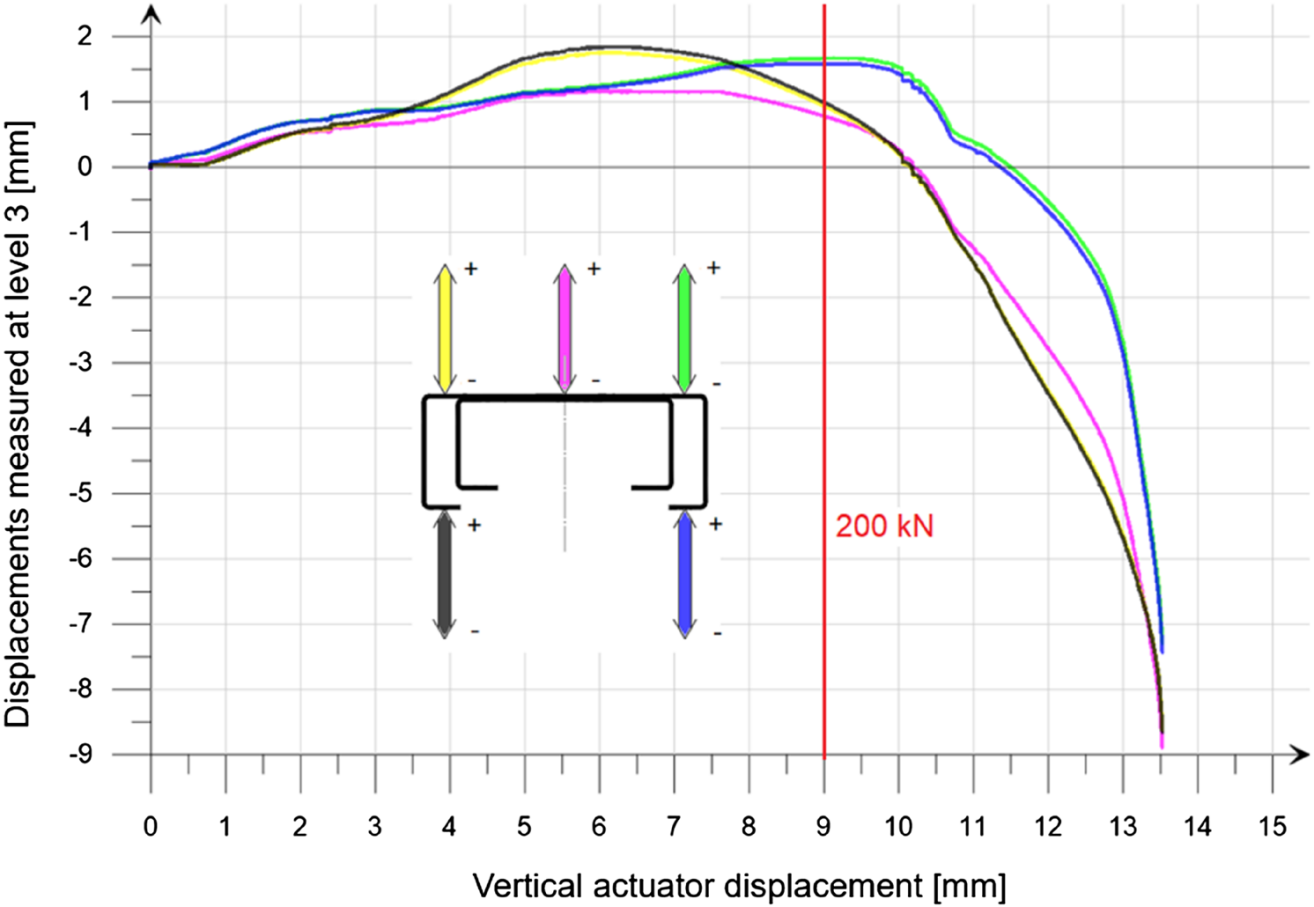
Level 3 displacements to the actuator vertical movement relationship for the column no. 6 (variant β).

On the measurement level 3, five displacement sensors were placed to measure the global deformation of the element and possible twisting of the column cross-section (Fig. 7b). A relationship diagram of the level 3 displacements to the actuator vertical movement for representative column is shown in Fig. 12. A change in the relationship between the applied force and the vertical displacement was observed, and it is visible in Fig. 9 as a graph perturbation between 8 and 10 mm of actuator displacement.

The red vertical line marked in Fig. 12 represents the actuator displacement corresponding to the load of 200 kN. In the force range from 0 to about 200 kN, no significant horizontal displacements were recorded. After exceeding the force of over than 200 kN, a change in the nature of the deformation was observed. The increase in global deformation (not exceeding 10 mm) at the time of failure was observed. No significant twisting or deformation of the cross-section was observed during the entire measurement.

It was noticed that the presence of the additional chord significantly inhibits the global deformation of the entire column in the positive direction relative to the axis marked in Fig. 12. Deformation in the negative direction is possible because the additional chord, at its top and bottom ends, does not need to rest on the main section.

## FEM analysis

### Modelling techniques

RFEM 6 software was used to perform the numerical FEM analysis. Both the geometry of the columns and the support conditions were modelled according to the experimental setup. The horizontal translations and rotation along the vertical axis were blocked in the column head. The head can rotate around the weaker axis. The last row of bolts was fixed to the vertical base plate. The lower edge of the main branch has been supported by restraining all translational and vertical rotational degrees. The thickness of the surface elements was assumed to be 3 mm. The contact between the surfaces of the sections was also taken into account in order to prevent the chords from penetrating each other during the deformation. Elastic friction and the possibility of mutual detachment of the surfaces were also taken into account (failure under tension perpendicular to surfaces). Friction coefficient between main and additional chord equal to 0.15 was assumed.

A non-linear model of plastic-elastic material was used in order to construct the thin-walled column chords, the description of which is presented in Sect. 3.1. Isotropic, plastic material model was used. Eight-point multilinear stress–strain diagram was defined according to obtained material test (Fig. 8a).

**Figure 13:**
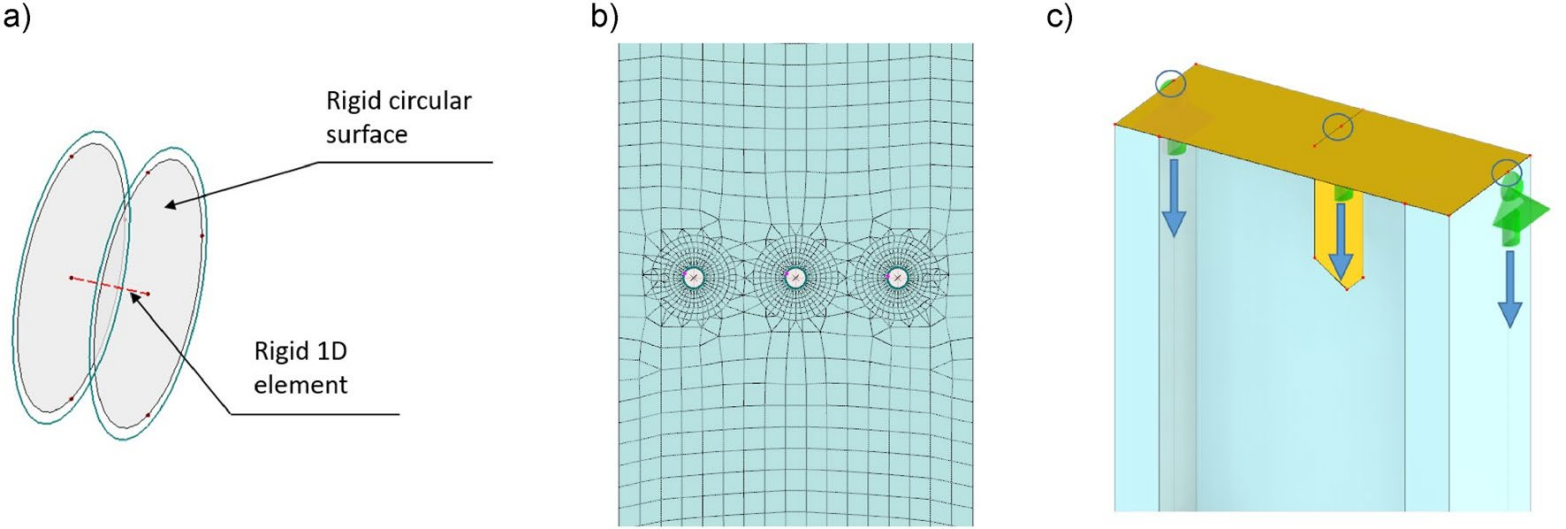
FEM model: (**a**) general view of simplified bolt connector, (**b**) mesh arrangement around bolts, (**c**) column head geometry and nodal supports location.

Due to the large difference in stiffness of the 3 mm cold-formed column steel sheet and the M12 bolt shank a simplified approach was used to model the bolt connectors. Additionally, visual inspection of the bolt connectors after experimental tests did not confirm any deformation or damage to the bolt shanks. Thus, the bolt connector was assumed to be rigid and consisted of two circular surfaces mutually connected by a rigid 1D element. Figure 13a shows the general view of the simplified connector.

The maximum length of the finite elements of both chords was assumed as 15 mm. FEM mesh density around the bolt connectors was increased. Circular mesh refinement zone with a radius of 75 mm was implemented. The inner element (within the zone) size was 5 mm and outer one was 15 mm. Figure 13b shows the mesh arrangement around bolts.

Reducing the size of the finite elements from 15 to 10 mm and 7 mm noticeably extended the calculation time, but did not significantly affect the results. A difference in the horizontal displacement of the column not exceeding 2% was noticed. It was decided to adopt a size of numerical elements equal to 15 mm for further calculations.

### Nonlinear FEM analysis results and model verification

In the first step, a model was created that takes into account the geometric and material non-linearity and the contact between the shells. The load was applied as a column head vertical support displacement. Figure 13c shows the column head geometry and the location of nodal supports. The line of supports coincides with the centre of gravity of the main chord. In order to find a solution to the non-linear problem, the Newton–Raphson method and the forced vertical displacement applied to the column head with a step of 0.10 mm were used. The head can rotate around the weaker axis. The results of the geometrically and materially non-linear analysis with contact (CGMNA) are presented in Table 5. The value of the maximum load was obtained by reading the reactions at points of application of the forced vertical displacement (Fig. 13c). In order to verify the FEM models and the results of the CGMNA analyses (RFEM 6), twin models were created in the Abaqus software. Comparing the results obtained from the two computational programs, a high consistency of the results was noticed (differences did not exceed 4%). Due to the ease of creating and modifying models in RFEM 6, it was decided to continue the analysis in this environment.

**Table 5:** Results of the CGMNA and CGMNIA analyses.

Analysed case	FEM type	FEM max force (kN)	Experiment max force* (kN)
α column *(test 1 − 3)*	CGMNA	365	320
	RFEM		
β column *(test 4 − 7)*	CGMNA	366	328
	RFEM		
α column *(test 1 − 3)*	CGMNIA	321	320
	RFEM		
β column *(test 4 − 7)*	CGMNIA	329	328
	RFEM		

*Rounded values of the median from Table 3.

In the next stage, a global bow imperfection was added in the form of column deformation along its entire length. The global imperfection was assumed in the plane of the least inertia of the cross-section with a maximum amplitude of 16 mm, which corresponds to the value of L/200^1,5^. The results of the CGMNIA analysis, taking into account imperfections, are presented in Table 5.

**Figure 14:**
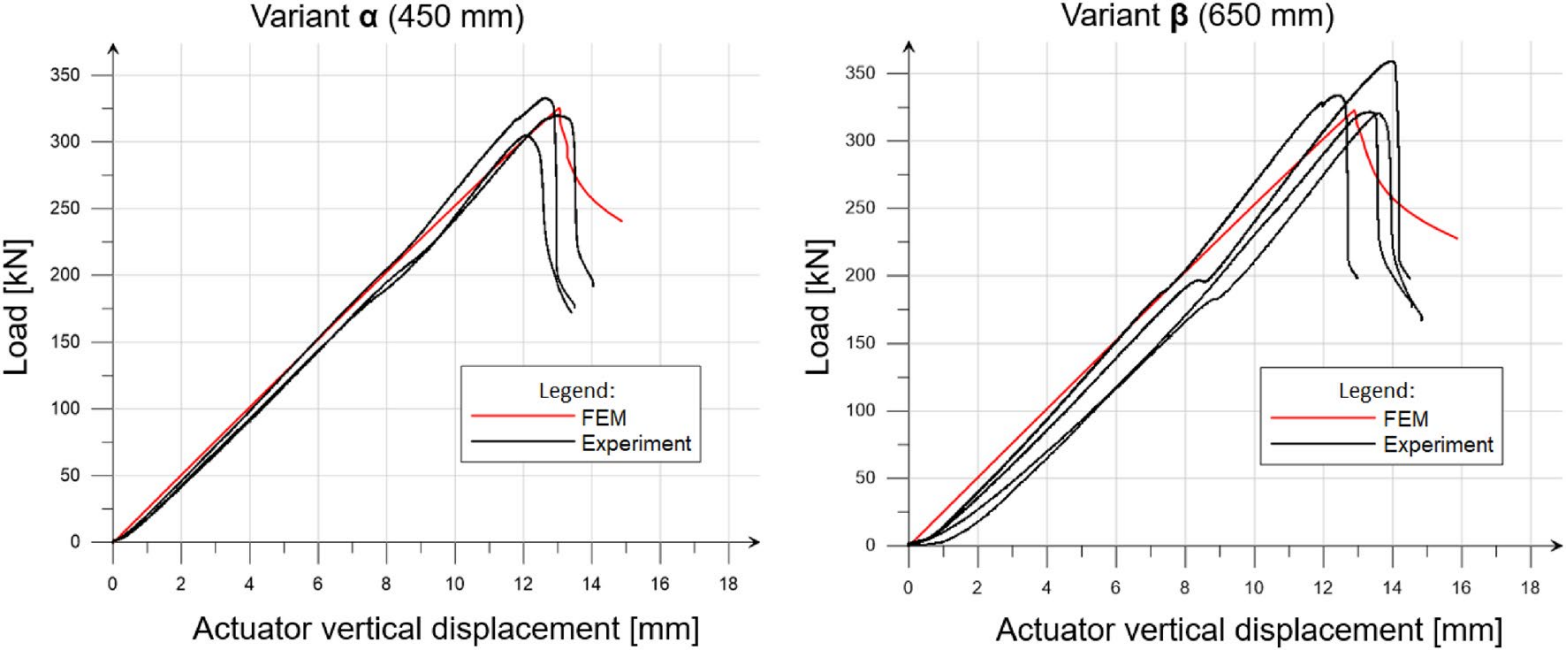
Load—actuator vertical displacement relationship.

Figure 14 shows the dependence of the column head vertical displacement on the actuator load—the static equilibrium path. The result of the numerical analysis (for the model with global imperfection) was compared with the experiment.

In the numerical model, the horizontal displacements in the middle of the height of the columns were controlled and compared with the values obtained in the laboratory experiment. At the moment of reaching the maximum force, the horizontal displacement was equal to 7.5 mm for FEM analyses and between 7 and 9 mm during experimental tests. The vertical displacements increase with the decrease of the load after reaching the ultimate load capacity was mainly a consequence of a global and distortional stability loss. Plastic deformation near the top series of bolt was also observed.

**Figure 15:**
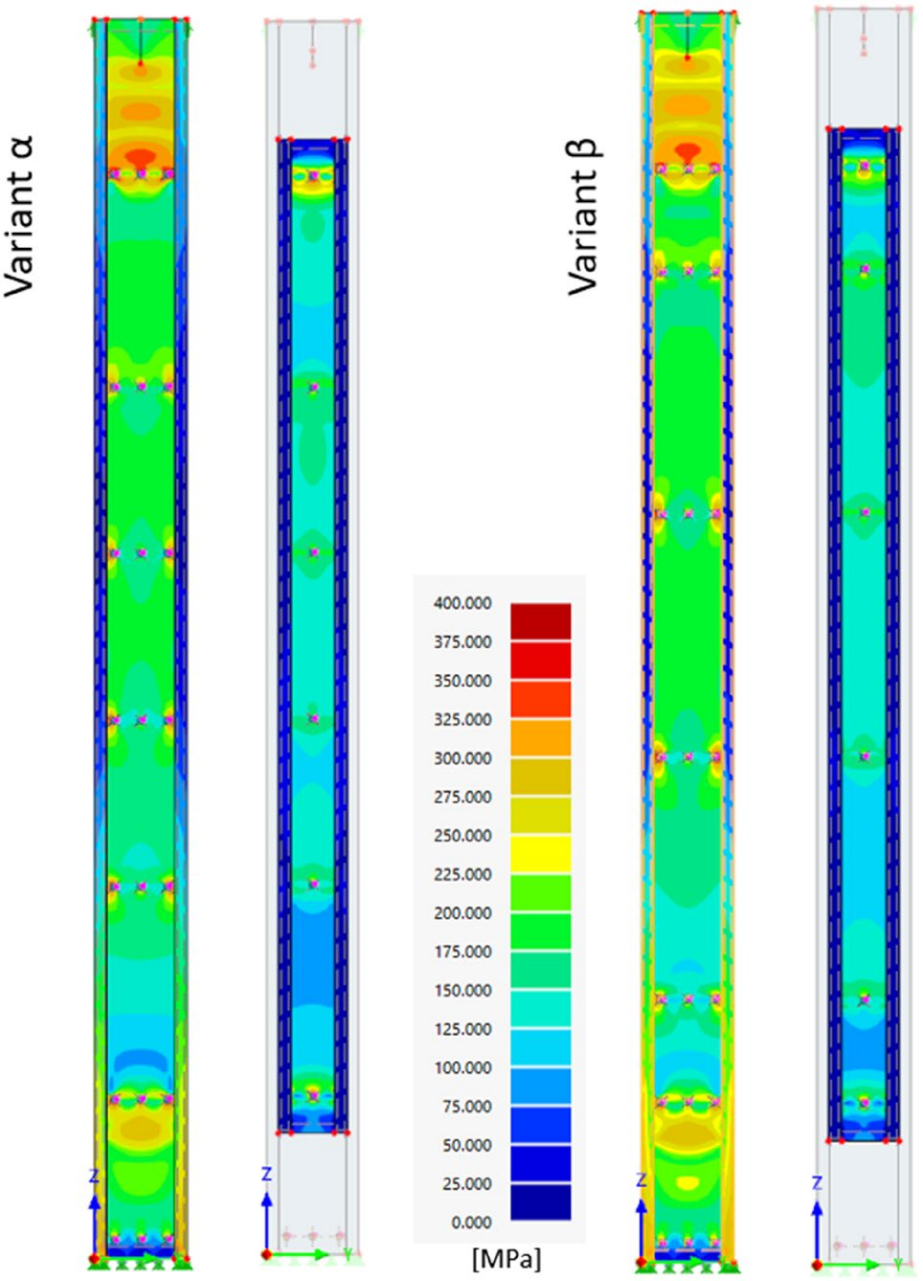
Distribution of Mises equivalent stresses [MPa] along the length of the main and additional chords—force of 300 kN.

Correspondence of the numerical results with the results obtained during the laboratory experiment was found. Figure 15 shows the distribution of Mises equivalent stresses along the length of the main chord and the additional chord obtained for both column variants during compression with a force of 300 kN.

Table 6 compares the stresses obtained from the experiment with the values of normal stresses measured along the column axis obtained from the FEM analyses. When reading the stresses from the numerical model, an extra care was taken to ensure that they corresponded to the side of the surface on which the strain gauges were located in the laboratory experiment.

**Table 6:** Comparison of normal stresses along the column axis obtained by experiment and FEM numerical analyses.

	Measurement level	10 kN		80 kN		160 kN		240 kN		300 kN	
		Inner chord	Main chord	Inner chord	Main chord	Inner chord	Main chord	Inner chord	Main chord	Inner chord	Main chord
		(MPa)									
α VARIANT											
Experiment	II	2	2	27	31	51	66	63	117	63	203
FEM	II	3	4	27	35	54	73	77	115	75	166
Experiment	III	3	3	31	31	60	61	92	97	114	124
FEM	III	4	4	31	34	62	69	94	104	118	128
Experiment	IV	4	3	30	32	51	62	65	113	58	203
FEM	IV	3	4	26	34	56	71	74	116	71	162
β VARIANT											
Experiment	II	1	1	18	22	43	53	66	97	69	171
FEM	II	4	4	29	35	58	72	87	107	109	133
Experiment	III	2	2	20	24	40	51	64	87	82	125
FEM	III	4	4	31	35	61	70	92	106	114	134
Experiment	IV	3	3	24	30	42	58	61	102	72	173
FEM	IV	3	4	28	35	56	72	84	105	105	129

It was noticed that in the force range from 10 to 240 kN, the normal stress values obtained in the laboratory experiment were slightly lower than those obtained numerically, but for the load level 300 kN experimental results are noticeably higher in the case of the main chord. Analysing Figs. 12 and 14, it can be seen that during the experimental test, when the force exceeds 250 kN, the direction of the global deformation of the column changes. The strain gauges on the main chord become additionally compressed which explains the stress increase. However, in the numerical model, global deformation was observed only in one direction during the entire load range.

### Additional FEM analyses

**Figure 16:**
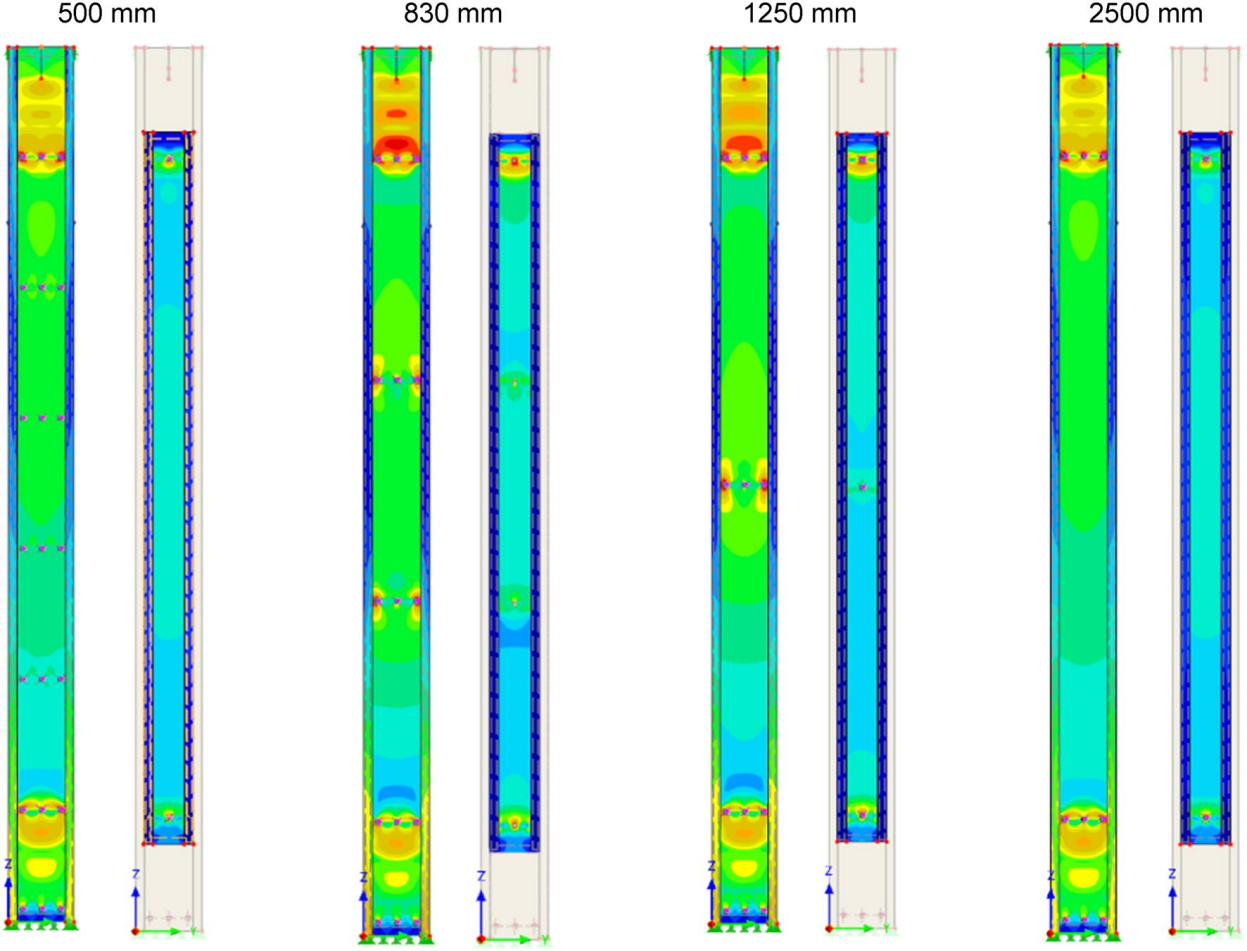
Mises equivalent stress distribution along both chords in four new variants for a force of 300 kN. The same stress scale applies in Figs. 15 and 16.

Satisfactory agreement of the FEM results with the laboratory experiment was the basis for further numerical analyses of subsequent column variants. In addition to the previously discussed built-up columns (α and β variants), extra numerical analyses of elements with series of bolts spacing equal to 500 mm, 830 mm, 1250 mm and 2500 mm were also carried out. The proposed additional variants were created based on the assumption of a constant series of bolt spacing along the length of the additional chord. Apart from changing the number of bolt series connecting both chords, the geometry of the columns remained unchanged (Fig. 3). Table 7 contains the results of additional FEM analyses. Figure 16 presents Mises equivalent stress distribution along both chords in four new variants for a force of 300 kN. The same stress scale applies in Figs. 15 and 16.

**Table 7:** Results of additional FEM analyses.

Interconnecting bolt spacing	Number of bolt series (rows) along the inner chord	Max FEM load (kN)
450 mm (acc. Figure 3 and 15)	6	321
650 mm (acc. Figures 3 and 15)	6	329
500 mm (acc. Figure 16)	6	322
830 mm (acc. Figure 16)	4	319
1250 mm (acc. Figure 16)	3	317
2500 mm (acc. Figure 16)	2	317

It was noticed that the change in the bolt spacing and the reduction in the number of bolts connecting the main chord with the internal chord slightly affected the maximum load capacity of the analysed structure. The highest load-bearing capacity was obtained when the number of bolts was concentrated in the zone associated with the beginning and end of the internal chord (β variant—Fig. 3). This relationship was noticed both in the laboratory experiment and numerical analyses.

## Summary

An experimental and numerical investigation on normal stress distribution in built-up cold formed columns in relation to interconnecting bolt spacing was presented in this paper. Seven built-up columns were tested experimentally distinguishing two variants of bolts spacing. First variant was based on Eurocode^5^ regulation. Columns with increased spacing by more than 40% was also tested. During each test stress distribution and load-bearing capacity were monitored. Additionally, column consisting only of main chord was also analysed experimentally. Numerical models were prepared and covered 6 bolt series layouts along the internal chord (including 2 experimental variants).

As expected, the presence of additional closely-spaced chord increased the maximum load-bearing capacity significantly. The average axial compression resistance of built-up column was 35% higher than single chord column (Table 3). This confirms the legitimacy of using this solution by the industry, when internal chord extension to the head and base plates is not possible.

The compiled stress distributions diagrams show, that the internal chord, despite its shorter length and lack of direct connection to the column head and base, takes a significant part of the stresses in the case of two experimentally tested variants.

Both, numerical and experimental analyses show that increasing the interconnecting bolt series spacing does not affect the bearing resistance significantly. Most efficient way of arranging the bolt series layout is to condense the number of bolts only in the very top and bottom area of the internal chord. In the case of future implementations of this type of construction, it is recommended to increase the number of fasteners at both ends of the internal chord and also to increase the spacing of the bolt rows in the middle area of the element.

An extension of the presented research will be an analysis of bolt series spacing in the context of anti-corrosion protection and the establishment of guidelines for contemporarily protected closely-spaced elements. Internal chord length influence on forms of stability loss and ultimate bearing capacity is also planned to examine in further research.

### Supplementary Information

Supplementary Information.

## Data Availability

Most of the data generated or analysed during this study are included in this published article and its supplementary files. The rest of the data will be available from the corresponding author on reasonable request.
